# Primary care usage at the end of life: a retrospective cohort study of cancer patients using linked primary and hospital care data

**DOI:** 10.1007/s00520-024-08458-7

**Published:** 2024-04-08

**Authors:** M. Grant, D. McCarthy, C. Kearney, A. Collins, V. Sundararajan, J. Rhee, J. Philip, J. Emery

**Affiliations:** 1 Palliative Nexus Research Group, Department of Medicine, University of Melbourne, Melbourne, Australia; 2 Department of Palliative Medicine, St Vincent’s Hospital Melbourne, Melbourne, Australia; 3 Centre of Expertise in Palliative Care Utrecht, Department of General Practice, Julius Centre, UMC Utrecht, Universiteitsweg 100, 3584CG Utrecht, The Netherlands; 4 Dept of General Practice and Primary Care, Centre for Cancer Research, University of Melbourne, Melbourne, Australia; 5 La Trobe University, Public Health, Melbourne, Australia; 6 Discipline of General Practice, School of Population Health, Faculty of Medicine and Health, UNSW Sydney, Sydney, Australia

**Keywords:** Palliative care, Primary care, End-of-life care, Home visits, Cancer

## Abstract

**Purpose:**

Health service use is most intensive in the final year of a person’s life, with 80% of this expenditure occurring in hospital. Close involvement of primary care services has been promoted to enhance quality end-of-life care that is appropriate to the needs of patients. However, the relationship between primary care involvement and patients’ use of hospital care is not well described. This study aims to examine primary care use in the last year of life for cancer patients and its relationship to hospital usage.

**Methods:**

Retrospective cohort study in Victoria, Australia, using linked routine care data from primary care, hospital and death certificates. Patients were included who died related to cancer between 2008 and 2017.

**Results:**

A total of 758 patients were included, of whom 88% (*n* = 667) visited primary care during the last 6 months (median 9.1 consultations). In the last month of life, 45% of patients were prescribed opioids, and 3% had imaging requested. Patients who received home visits (13%) or anticipatory medications (15%) had less than half the median bed days in the last 3 months (4 vs 9 days, *p* < 0.001, 5 vs 10 days, *p* = 0.001) and 1 month of life (0 vs 2 days, *p* = 0.002, 0 vs 3 days, *p* < 0.001), and reduced emergency department presentations (32% vs 46%, *p* = 0.006, 31% vs 47% *p* < 0.001) in the final month.

**Conclusion:**

This study identifies two important primary care processes—home visits and anticipatory medication—associated with reduced hospital usage and intervention at the end of life.

**Supplementary Information:**

The online version contains supplementary material available at 10.1007/s00520-024-08458-7.

## Introduction

The final year of a person’s life is often unpredictable, with loss of mobility and independence, and the many changing physical, social, psychological and existential impacts of deteriorating health [1]. These patients have many complex and evolving care needs, which may require the involvement of multiple different care systems and providers across hospitals, primary care, community palliative care and hospice teams [2]. The last 3 months of a person’s life are the most resource intensive in terms of health system usage, with approximately 80% of this expenditure occurring in hospital [3, 4]. Hospital admissions, emergency department visits and interventions such as surgery and chemotherapy may be of limited benefit for patients and deprive them and their family of time that may be otherwise spent in the home environment [5, 6]. However, care at home is often not possible, reliant on informal caregivers, the care environment and primary care support [7].

Primary care services and general practitioners (GPs) have a central role in the care of patients with palliative care needs, through addressing the patient’s varied concerns, providing day-to-day care including symptom management, collaborating with specialists and other care providers, providing psychosocial and existential support to the patient and family and coordinating the care team [8–10]. Close involvement of primary care services at the end of life for people with cancer has been promoted internationally as a manner to provide quality end-of-life care that is appropriate to the needs of patients, and may reduce unnecessary interventions, hospitalisations and health care expenditure [1, 2, 11–14]. This role is of increasing importance given the worldwide ageing population, the consequent number of people who will require end-of-life care and the required responses of health systems to meet these needs [11].

Despite widespread acknowledgement of the effectiveness of close primary care involvement at the end of life, the extent of this involvement varies considerably [14–16]. In many countries such as Australia, involvement of primary care appears to shift throughout the illness course [16]. Prior to the terminal phase, patients with cancer frequently receive intensive hospital-based treatments such as chemotherapy for extended periods, when primary care may have limited involvement [17]. When these patients’ cancer progresses, there is often a shift back to primary care, although a number of patients may continue to be managed exclusively by specialist teams or die suddenly in hospital [16]. The use and patterns of primary care for cancer patients at the end of life in Australia and internationally are not well understood, nor is the relationship between primary care involvement and hospital use.

This study aims to (i) describe the use of primary care services at the end of life, and (ii) identify how this care, or key care processes, are related to the use of hospital care services.

## Methods

### Study design

This study was designed as an observational cohort study incorporating routine clinical and administrative data from the state of Victoria, Australia. This study is reported in line with the Strengthening the Reporting of Observational Studies in Epidemiology (STROBE) Statement [18]. This research was reviewed and approved by the institutional review board of St Vincent’s Hospital Melbourne (LLR 074/19) and the National Prescribing Service Medicine Insight Data Governance Committee (2020–013).

### Data sources

This study incorporated linked data from five different datasets, linked through Biogrid Australia. National Prescribing Service MedicineWISE MedicineInsight dataset includes longitudinal data from primary care clinics as part of routine clinical care, incorporating approximately 10% of Australian primary care clinics [19]. The National Death Index is a national dataset describing all Australian deaths containing person level records of all deaths. Hospital, emergency and outpatient data were collected from three datasets: The Victorian Admitted Episodes Dataset, the Victorian Emergency Minimum Dataset and the Victorian Integrated Non-Admitted Health dataset, from two large health services in metropolitan Melbourne and its regional surroundings, incorporating six hospitals and outpatient facilities. These datasets include de-identified demographic, clinical and administrative details, including clinical diagnoses, admissions, transfers and clinical care processes such as surgery, chemotherapy, radiotherapy and imaging.

### Setting and population

The data included patients from Victoria, Australia (population 6.7 million in 2020), who died of a cause likely to be related to cancer between 1 July 2008 and 31 December 2017. Inclusion criteria included over the age of 18 at death, at least one primary care encounter in the 12 months prior to death, a coded diagnosis of cancer in the hospital dataset in the 2 years prior to death, and who died from a cause likely related to their cancer diagnosis. A death likely related to a cancer diagnosis was defined as those people diagnosed with metastatic or poor prognosis cancers (see supplementary file table A), or for whom cancer was listed as a cause of death. Patients were excluded who resided outside of the catchment areas of the health services, resulting in a largely metropolitan population.

### Data

Patients were selected who had a record of cancer in the hospital datasets. Patients were selected from the National Death Index who had a registered date of death from 1/07/2008 to 31/12/2017, to allow for 1 year of MedicineInsight data and 2 years of hospital data prior to death. Linkage then proceeded between all datasets.

Data was collected from the hospital datasets describing patient demographics (age, area of residence, sex), clinical characteristics and emergency department, hospital and specialist care provision. Cancer diagnoses and other major comorbidities were identified based on the International Statistical Classification of Diseases and Related Health Problems (10th edition), Australian Modification codes [20, 21]. Previously described indicators of inappropriate end-of-life care as described by Earle and de Schreye were collected through standardised clinical procedure codes at different time points, including surgery, imaging, laboratory testing, intensive care (ICU) and chemotherapy [22, 23].

Variables describing primary care provision included number and types of contacts, medication prescribed and requests for laboratory and imaging tests. Contacts were defined as episode of care provision, which involved the GP or nurse, and were not purely administrative. This included contacts that were not billable, including telephone contact with patients, caregivers and specialists, and prescribing medications. Home visits were identified through Medicare Benefits Schedule billing codes and free text describing the reason for consultations. Anticipatory medications were defined as the proactive prescribing of injectable medicines that are commonly required to control symptoms in palliative care, detailed in supplementary file table B and based on existing literature [24].

### Data analysis

Patient demographics and clinical characteristics were detailed using descriptive statistics. Dates of admissions, consultations, investigations, prescribing and interventions were all employed to calculate the time between the event and the death of the patient, and grouped in monthly intervals according to their relation to the date of death. Calculations were expressed as means and standard deviations for parametric data, and medians and interquartile ranges for non-parametric distributions. Chi-squared testing was employed for comparisons of difference for categorical variables, and Mann–Whitney *U* or Kruskal–Wallis tests for non-parametric data. For the patterns of care data, a negative binomial model was estimated using the number of bed days as the outcome, age as a continuous variable and type of primary care user (irregular, regular or high user) as the variable of interest. A *p* value of less than 0.05 was considered statistically significant. Linkage was performed utilising Stata 15 (Stata Corp, 2017, College Station, TX, USA). IBM SPSS software version 27 (IBM, 2021, Chicago, IL, USA) was employed for data analysis.

## Results

A total of 40,881 patients were identified with a cancer diagnosis in the hospital datasets. After excluding patients that did not meet the inclusion criteria, a total of 758 patients were included in this study, described in supplementary file figure A.

### Patient characteristics

Of the 758 included patients, 339 (45%) were female, with a mean age of 70.6 years, described in Table 1. The most common cancer types were lung (21%), upper gastro-intestinal (18%) and lower gastrointestinal (13%) tumours, with 71% of patients having metastatic disease.

**Table 1:** Patient clinical characteristics

		*N* = 758 (%)
Sex	Female	339 (45)
Age—mean (SD)		70.6 years (13.9)
Cancer type	Lung	162 (21)
	Upper gastrointestinal	134 (18)
	Lower gastrointestinal	100 (13)
	Haematological	85 (11)
	Genito-urinary	76 (10)
	Breast	51 (7)
	Head and neck	28 (4)
	Gynaecological	26 (3)
	Skin	26 (3)
	Neurological	14 (2)
	Unknown primary / other	56 (7)
Metastatic disease		541 (71)
Major comorbidity	Renal failure	115 (15)
	Heart failure	95 (13)
	Chronic obstructive pulmonary disease	72 (10)
	Dementia	24 (3)

### Usage of primary care services

**Fig. 1:**
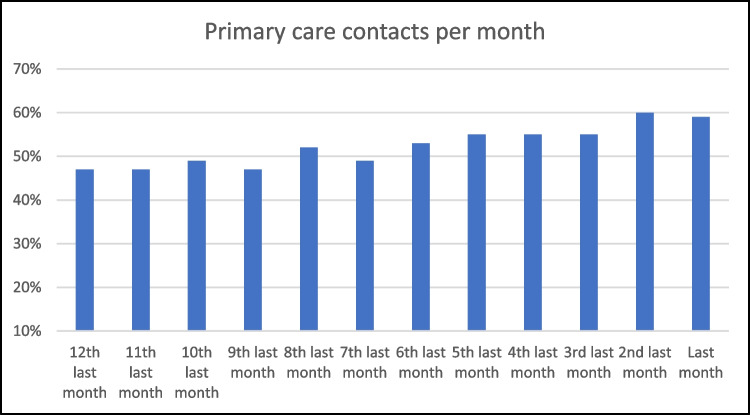
Proportion of patients accessing primary care for each month prior to death

Patients had a median 14 primary care episodes in the last year of life. The majority of patients had primary episodes in the last 6 (88%), 3 (77%) and 1 (59%) month of life, as described in Table 2. Figure 1 describes the timing of these episodes, with 46–52% of patients engaging with primary care between 12 and 7 months prior to death, and this percentage increasing towards the end of life, with 60% of patients accessing primary care in the second-last month of life, and 59% in the month prior to death.

**Table 2:** Primary care service use in the last year of life

	Time period	
Number of primary care contacts	Last 12 months—med [IQR]	14 [5–24]
	6 months—med [IQR]	8 [2–15]
	3 months—med [IQR]	4 [1–8]
Patients who had contact with primary care	Last 6 months—*n* (%)	667 (88)
	3 months—*n* (%)	585 (77)
	1 month—*n* (%)	447 (59)
Home visit	Last 3 months—*n* (%)	95 (13)
	1 month—*n* (%)	73 (10)
Care processes		
- Opioids prescribed	Last 3 months—*n* (%)	341 (45)
- Anticipatory medications	Last 3 months—*n* (%)	115 (15)
- Imaging	Last month—*n* (%)	23 (3)
- Laboratory tests	Last 2 weeks—*n* (%)	28 (4)

Relatively few patients received home visits (13%) or anticipatory medications (15%), and 341 (45%) were prescribed opioids in the last 3 months of life. The proportion of patients who received potentially inappropriate treatments at the end of life as described by de Schreye was low, including imaging (3%) in the last month, and laboratory tests (4%) in the last fortnight [22].

### Patterns of primary care use

Exploratory analysis identified three cohorts based on the regularity and quantity of primary care contacts: patients who accessed primary care on an irregular basis (*n* = 302, 40%) [irregular users], patients who regularly accessed primary care in normal quantity (*n* = 282, 37%) [regular users] and patients who accessed care regularly and in high quantity (*n* = 174, 23%) [high users]. Regularity was defined by primary care contacts every 4 months over the last 12 months of life, and high quantity defined by greater than the p75 for number of primary care contacts.

Irregular users had a median age of 67 years and were the youngest cohort compared to regular (71.5 years) and high users (75.0 years). Hospital admissions, bed days, emergency presentations and use of other hospital care services did not differ between cohorts. This is described in supplementary file, table C.

### Home visits and anticipatory medications

**Fig. 2:**
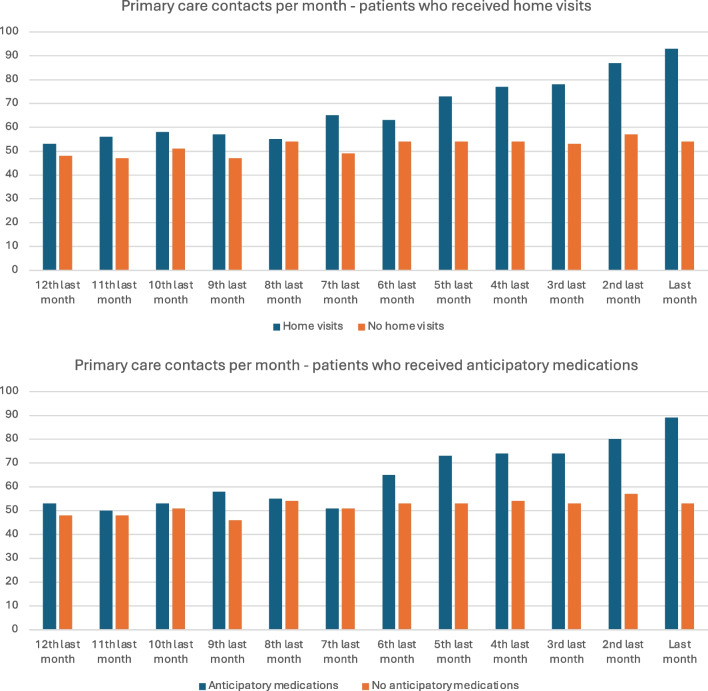
Proportion of patients accessing primary care for each month prior to death, according to access to home visits and anticipatory prescribing

An exploratory analysis of primary care identified two variables or care processes that were associated with different usage of hospital care: home visits and prescribing of anticipatory medications. Ninety-five patients (13%) received one or more home visits in the last 3 months of life, and 133 (15%) were prescribed one or more anticipatory medications, with 45 patients having both. Table 3 describes usage of primary and hospital care for these patients, and Fig. 2 describes the patterns of primary care contacts per month.

**Table 3:** Patient primary and hospital service use according to receipt of home visits and anticipatory medications

	Home visits			Anticipatory medications		
	Yes	No	*p* value	Yes	No	*p* value
Number patients	95	663	-	133	625	-
Age (mean)	75.9	69.8	-	70.6	70.6	-
Primary care contacts—med [IQR]						
Month 7 to 12	7 [1–14]	5 [1–10]	0.017	6 [1.5–12]	5 [ 1–10]	0.085
Month 4–6	6 [2–10]	3 [0–6]	< 0.001	5 [2–8]	3 [0–7]	< 0.001
Last 3 months	11 [7–16]	3 [1–7]	< 0.001	8 [5–13]	3 [0–7]	< 0.001
Hospital bed days—med [IQR]						
3 months	4 [0–12]	9 [1–22]	< 0.001	5 [0–15]	10 [1–22]	0.001
1 month	0 [0–5]	2 [0–5]	0.002	0 [0–4]	3 [0–12]	< 0.001
Hospital care service use in last month of life—% patients						
Hospital admission	47%	61%	0.010	47%	61%	0.002
Emergency department presentation	32%	46%	0.006	31%	47%	< 0.001
Surgery	5%	7%	0.344	2%	8%	0.010
ICU	2%	6%	0.101	0%	6%	0.004
Chemotherapy	2%	5%	0.193	4%	4%	0.465
Imaging	2%	5%	0.211	2%	5%	0.059

The 95 patients who received home visits had an older mean age of 75.9 years (vs 69.8 years). These patients had double the primary care episodes between 4 and 6 months prior to death (median 6 vs 3, *p* < 0.001) and almost four times as many episodes in the last 3 months of life (median 11 vs 3, *p* < 0.001). Patients who received home visits had significantly fewer hospital bed days in the last three (median 4 vs 9 days, *p* < 0.001) and month (median 0 vs 2 days, *p* = 0.002) of life. Fewer patients in this cohort had hospital and emergency department admissions in the last month of life, but there were no differences in the rates of ICU admissions, surgery, chemotherapy or imaging.

Anticipatory medications were prescribed to 133 patients, whose mean age was identical to those patients who did not receive them (mean 70.6 years). The most commonly prescribed medications were morphine and metoclopramide, received by 123 (92%) and 105 (78%) patients respectively. The most common combination of anticipatory medications was opioids and antiemetics, prescribed to 86 (65%) of patients. Further description of anticipatory prescribing is detailed in the appendices, Table D. These patients also had similar patterns of accessing primary care from month 7 to 12 prior to death, (median 6 vs 5 episodes, *p* = 0.085), which intensified in the last 3 months of life (median 8 vs 3, *p* < 0.001). Patients prescribed anticipatory medications had significantly fewer bed days in the last 3 months (median 5 vs 10 days, *p* = 0.001) and last month (median 0 vs 3 days, *p* < 0.001) of life. Fewer patients in this cohort had hospital or ICU admissions, accessed emergency departments and had surgery at the end of life.

## Discussion

### Main findings/results of the study

This study describes the important role of primary care at the end of life, with 88% of patients accessing primary care services in the last 6 months of life, and this care increasing in intensity in the months prior to death. This care pattern appears to reflect the response to the increasing care needs of cancer patients as they approach the end of life, with small proportions of patients receiving potentially inappropriate care (such as imaging and laboratory tests) through primary care at the end of life [14]. These findings reflect the use of primary care services at the end of life in other countries, such as described Gao et al. in England [25]. The results highlight two primary care processes associated with decreased use of hospital care services: home visits and anticipatory prescribing. This is an important finding, suggesting that primary care service delivery is an important component of enabling patients to remain at home, and avoiding unnecessary hospitalisations and emergency presentations at the end of life.

### What this study adds

Whilst an association exists, these results should not be interpreted that home visits and anticipatory prescribing cause reductions in hospital care usage. Primary care is the result of many longitudinal and frequently highly complex care processes, of which the prescription of injectable medications or home consultation is embedded within the continuity of care [26]. Rather, they may be better viewed as a reflection, or indicator, of quality primary care provision at the end of life. Patients who accessed these services received more intensive primary care involvement in the months prior to death, especially the last 3 months of life when their care needs were likely greatest, reflecting care that was reactive to care needs. It is possible that this cohort who received these interventions were subject to selection bias, as they were patients who were wished to remain at home, and thus more likely to receive home visits and anticipatory medications [27]. They may also have been patients with a more predictable prognostic course. However, this again may be a signifier of quality care, of primary care physicians and nurses who possessed the skill and experience to identify the care needs of their patients, discuss their advance care plans and enact these care processes in anticipation of their terminal decline [10].

Home visits have been identified by previous research as being associated with quality end-of-life care outcomes, including increased probability of dying at home and decreased emergency department usage [13, 28, 29]. Our results add further weight to these findings, also describing an association with reduced hospital admissions and bed days. Despite this, few patients were able to access home visits, with previous research highlighting barriers of poor renumeration, time restraints and lack of confidence in providing home palliative care [30, 31]. In Australia, government funding for general practices to conduct home visits is limited, and unless patients are able and willing to pay privately, GPs experience a significant financial loss in providing this service. There is relatively limited evidence regarding anticipatory prescribing, with only a single small study examining health service usage, which identified reduced hospital admissions for those patients prescribed anticipatory medications [32]. It is a practice employed in a minority (14–16%) of patients, with substantial variations in use and safety issues that require further evaluation to guide best practice [24, 27].

The recent study of Leniz et al. demonstrated that patients with higher usage of primary care services are more likely to have multiple admissions and emergency department presentations [33]. However, the concept of ‘longitudinal continuity of care’, which measures the regularity of primary care episodes over time, has not been applied to palliative care [34, 35]. One of the hypotheses that drove this research was to investigate the association between continuity of primary care use and hospital service usage. For patients with chronic diseases such as diabetes, patients who regularly access primary care are less likely to have unplanned admission and emergency department presentations than irregular users [34, 36]. Whilst there were different patterns of use evident in our results, this did not translate to noticeable differences in outcomes. This may be related to the heterogenous nature of the population, which included patients with different cancer types, whose disease trajectories and care needs may have varied substantially. Further research focused on singular cancer types or patient subgroups would be required to explore this further.

### Strengths and weaknesses/limitations of the study

This study utilises linked retrospective datasets from five differing datasets, comprising administrative, clinical and billing data, providing a broad understanding of the demographic and clinical characteristics of the patients, and the clinical procedures and processes that were employed in primary and hospital care. Previous studies examining primary care use at the end of life have largely employed billing or health insurance data, which provides limited insight into exactly what care is provided by primary care. When identifying the cohort for this study, we employed restrictive inclusion criteria to only include those patients who (i) were active patients of the primary care practice and hospital service and (ii) died likely related to a cancer diagnosis. In the Australian health care system, patients are not registered to a single primary care practice or hospital and may visit multiple GPs and hospitals in the last year of life. The MedicineInsight data includes data for approximately 10% of primary care patients. Through utilising a strict inclusion criteria, we aimed to focus only on those patient who died of their cancer and whose health service usage we could reliably determine; however, this led to large numbers of patients excluded from this study.

Utilising linked retrospective datasets has limitations. The numbers included in this study may appear limited compared to the population sampled, which is related to three separate linkage processes and the strict inclusion criteria employed in the study. Yet these numbers were sufficient to identify care processes associated with reduced hospital usage, however, were not sufficient for further sub-group analyses. A number of valuable variables could not be obtained due to legal restrictions through the data linkage process or lack of available data, such as place of death and community palliative care services, which would have added further granular detail to this study. Additionally, important indicators such as ethnicity and socio-economic status are not available in these data. This cohort contains a metropolitan region of Australia and cannot be generalised to rural and remote regions. Whilst these data are from 2008 to 2017, our focus is the patterns of health service usage which has not altered significantly in this time.

## Conclusion

Primary care is central to end-of-life care for cancer patients, providing care that is largely accessible, responsive, with a low prevalence of potentially inappropriate care. The majority of patients in this study accessed primary care in the last 6 months of life, with the intensity increasing towards the end of life. This study identified two care processes associated with decreases in hospital service use—anticipatory prescribing and home visits—which may reflect a continuum of appropriate care processes in primary care.

### Supplementary Information

Below is the link to the electronic supplementary material.

Supplementary file1 (DOCX 99 KB)

## Data Availability

This work has utilised routine clinical and administrative data on an individual level, containing information that could compromise the privacy of participants. The data are thus not publicly available. The data may be made available upon reasonable request, dependent on their use, with enquiries directed towards the corresponding author (M Grant).
