# Correction to: Primary care usage at the end of life: a retrospective cohort study of cancer patients using linked primary and hospital care data

**DOI:** 10.1007/s00520-024-08712-y

**Published:** 2024-07-11

**Authors:** M. Grant, D. McCarthy, C. Kearney, A. Collins, V. Sundararajan, J. Rhee, J. Philip, J. Emery

**Affiliations:** 1 Palliative Nexus Research Group, Department of Medicine, University of Melbourne, Melbourne, Australia; 2 Department of Palliative Medicine, St Vincent’s Hospital Melbourne, Melbourne, Australia; 3 Centre of Expertise in Palliative Care Utrecht, Department of General Practice, Julius Centre, UMC Utrecht, Universiteitsweg 100, 3584CG Utrecht, The Netherlands; 4 Dept of General Practice and Primary Care, Centre, for Cancer Research, University of Melbourne, Melbourne, Australia; 5 La Trobe University, Public Health, Melbourne, Australia; 6 Discipline of General Practice, School of Population Health, Faculty of Medicine and Health, UNSW Sydney, Sydney, Australia

**Correction to: Supportive Care in Cancer (2024) 32:273**

10.1007/s00520-024-08458-7

The published paper has the incorrect Electonic Supplementary Materials and is now corrected.

### Supplementary Information

Below is the link to the electronic supplementary material.

Supplementary file1 (DOCX 89 KB)

