# Supplementary material for: Primary care usage at the end of life: a retrospective cohort study of cancer patients using linked primary and hospital care data
Source: Support Care Cancer. 2024 Apr 8;32(5):273. doi: 10.1007/s00520-024-08458-7 (PMC11001688; doi:10.1007/s00520-024-08458-7)
Supplement: Supplementary file 1 — Supplementary file1 (DOCX 99 KB) [file 520_2024_8458_MOESM1_ESM.docx]

**Supplementary Files**

| - - - Mesothelioma (C45) |
| --- |
| - - - Pancreas (C25) |
| - - - Unknown primary (C26, C39, C76, C80) |
| - - - Acute lymphoblastic leukaemia [ALL] (C91.0) where patient >65 years old at diagnosis |
| - - - Lung (C34) |
| - - - Acute myeloblastic leukaemia [AML] (C92.0) |

**Table A – Poor prognosis cancer and ICD-10 codes**

| **Active medication** | **Form** |
| --- | --- |
| Morphine | ampule for injection |
| Fentanyl | ampule for injection |
| Oxycodone | ampule for injection |
| Hydromorphone | ampule for injection |
| Metoclopramide | ampule for injection |
| Cyclizine | ampule for injection |
| Haloperidol | ampule for injection |
| Midazolam | ampule for injection |
| Clonazepam | ampule for injection |
| Glycopyrrolate | ampule for injection |

**Table B – Anticipatory medications**

**Figure A –** Flow diagram describing patient inclusion.

| **Type user#** | **Irregular** | **Regular** | **High** |
| --- | --- | --- | --- |
| Number patients | 302 | 282 | 174 |
| Age (mean) | 67.2 | 71.5 | 75.0 |
|  |  |  |  |
| Hospital Bed days – med [IQR] |  |  |  |
| 6 months | 12 [3-26] | 14 [4-32] | 12 [3-25.3] |
| 3 months | 6 [1-9] | 10 [1-23] | 9.5 [1-20] |
| 1 month | 2 [0-9] | 2.5 [2.5-11] | 1 [0-10.25] |
| Hospital Admission - % patients |  |  |  |
| 3 months | 78% | 78% | 77% |
| 1 month | 60% | 60% | 57% |
| Emergency department - % patients |  |  |  |
| 3 months | 65% | 63% | 65% |
| 1 month | 45% | 44% | 45% |
| Hospital care services - % patients |  |  |  |
| Surgery last 3 months | 12% | 16% | 13% |
| ICU last 3 months | 7% | 7% | 7% |
| Chemotherapy last month | 4% | 5% | 3% |
| Imaging last month | 5% | 4% | 3% |
|  |  |  |  |

# Irregular users defined as not having regular primary care contact every four months in the last year of life, Regular as having contacts at least once per four months in the last year of life, High users as having contacts at least once per fours months, and greater than 25 contacts in the last year of life.

*statistical testing of differences was conducted (with negative binomial regression to correct for age) and demonstrated no differences, thus is not presented.

**Table C –** Patient characteristics and hospital care use on basis of longitudinal continuity of care cohort.

|  | Number of patients prescribed (n=133) |
| --- | --- |
| Medication |  |
| - Morphine | 123 (92) |
| - Metoclopramide | 104 (78) |
| - Midazolam | 19 (14) |
| - Hydromorphone | 5 (4) |
| - Fentanyl | 3 (2) |
| - Clonazepam | 3 (2) |
| - Glycopyrrolate | 2 (2) |
|  |  |
| Anticipatory medication combinations prescribed* |  |
| - Opioids alone | 22 (17) |
| - Benzodiazepines alone | 1 (1) |
| - Antiemetics alone | 3 (2) |
| - Opioids + benzodiazepines | 5 (4) |
| - Opioids + antiemetics | 86 (65) |
| - Benzodiazepines + antiemetics | 1 (1) |
| - Opioids + benzodiazepines + antiemetics | 15 (11) |

*given the low numbers of patients prescribed anti-secretory medications, these were not included

**Table D –** Anticipatory medication prescribing
